# Anomaly Detection Based Latency-Aware Energy Consumption Optimization For IoT Data-Flow Services

**DOI:** 10.3390/s20010122

**Published:** 2019-12-24

**Authors:** Yuansheng Luo, Wenjia Li, Shi Qiu

**Affiliations:** 1 School of Computer and Communication Engineering, Changsha University of Science and Technology, Changsha 410114, China; 2 Department of Computer Science, New York Institute of Technology, New York, NY 10023, USA; 3 School of Economics and Management, Changsha University, Changsha 410022, China; 121601049@csu.edu.cn

**Keywords:** internet of things, fog computing, E-Health monitoring system, anomaly detection, latency awareness, energy efficient, mixed integer nonlinear programming

## Abstract

The continuous data-flow application in the IoT integrates the functions of fog, edge, and cloud computing. Its typical paradigm is the E-Health system. Like other IoT applications, the energy consumption optimization of IoT devices in continuous data-flow applications is a challenging problem. Since the anomalous nodes in the network will cause the increase of energy consumption, it is necessary to make continuous data flows bypass these nodes as much as possible. At present, the existing research work related to the performance of continuous data-flow is often optimized from system architecture design and deployment. In this paper, a mathematical programming method is proposed for the first time to optimize the runtime performance of continuous data flow applications. A lightweight anomaly detection method is proposed to evaluate the reliability of nodes. Then the node reliability is input into the optimization algorithm to estimate the task latency. The latency-aware energy consumption optimization for continuous data-flow is modeled as a mixed integer nonlinear programming problem. A block coordinate descend-based max-flow algorithm is proposed to solve this problem. Based on the real-life datasets, the numerical simulation is carried out. The simulation results show that the proposed strategy has better performance than the benchmark strategy.

## 1. Introduction

The promising big data applications based on IoT produced so much data [1,2], and thus it is impractical to transfer all these data to the data center for processing in real time. To address these challenges, the fog computing and edge computing are proposed in recent years as the distributed cloud computing solution for IoT applications. Due to the limited computation and communication capability of IoT end devices, some extensive computing models should be provided for processing large amount of IoT data. Fog computing is one of promising technologies that provide computation and communication services to IoT applications [3,4]. The concept of fog computing is similar to the edge computing [5,6,7,8,9]. Both of them are devoted to provide computation and communication resources for IoT users in the proximate area of IoT devices. According to the definition of European Telecommunications Standards Institute (ETSI), Multi-access Edge Computing (MEC) is one of the key technologies towards 5G and characterized by several merits such as ultra-low latency, high bandwidth, location awareness, etc. [5]. The edge computing nodes (MEC nodes) are deployed at many locations with access points, such as at the macro LTE base stations, at a multi-Radio Access Technology cell aggregation site, etc. [5]. On the other side, the fog computing nodes are not necessarily deployed around these access points. To distinguish these two concepts, in this paper, nodes with sufficient communication and computing resources scattered among IoT devices are referred to as **fog nodes**. The nano-server clusters deployed at the access points are referred to as **MEC nodes**, which usually have more resources than fog nodes. The collaborative computing of the IoT-end nodes, the fog nodes, the MEC nodes and the data center are referred to as **IoT-Fog-Edge computing** in this context.

The studies on collaboration of Fog, MEC, and Cloud computing usually focus on finding an optimal solution to allocate the IoT tasks to appropriate virtual machines which are hosted on fog, MEC or cloud nodes. In [10], the authors propose to allocate the workload among local MEC servers, neighborhood MEC servers or cloud servers to minimize the energy consumption of MEC nodes subject to delay constraints. Lyapunov drift-plus-penalty-based dynamic queue evaluation is used for the online allocation algorithm. In [11], an optimal algorithm is put forward to determine that the tasks should be allocated to clouds near the end devices or to the one far from the end devices for the energy efficient big data processing. The delay constraints to tasks in near clouds and far cloud are taken into account. In [12], an optimal algorithm for joint task allocation among mobile devices, the computing access point and the remote cloud is proposed, where computing access point can be treated as an MEC node. The studies introduced above are not correlated with the continuous data-flow (CDF) problem, which is the main concern of our work. The CDF is the data-flow continuously generated by IoT end nodes and is to travel through the fog nodes and MEC nodes before it reaches the cloud data center. The optimization to CDF problem is an optimization in multi-stage graph. On the contrary the optimization problem introduced in above studies is one-stage optimization problem. The typical CDF application in IoT-Fog-Edge computing is the E-Health Monitoring System [13,14,15,16], which will be elaborated as the Motivation Scenario in section II. Some efforts were devoted to the performance measurement and optimization of E-Health system [13,14,15,16] from the perspective of architecture and deployment optimization. To the best of our knowledge, there is little work required to optimize the performance of E-Health monitoring system from the perspective of mathematical modelling and programming.

To optimize the energy consumption of IoT devices while subject to the latency constraints, the anomaly should be discovered because the anomalous nodes would cause abnormal latencies and job loss or task failure on transmission paths [17,18]. As a result, additional retries and retransmissions will occur, resulting in increased energy consumption. Anomaly detection is a conventional method in the wireless sensor networks (WSN) [19,20,21,22], which is organized using the ad hoc way. The ad hoc mechanism makes the whole WSN system vulnerable to the intrusion of malicious nodes, thus the anomaly detection is carried out for finding the anomaly. The deficiency of anomaly detection in WSN is that many messages may be generated and exchanged in the networks. On the other hand, the network topology of fog and MEC computing is relatively stable and the nodes have more computation resources. Some systematic security and safe mechanism could be adopted in the IoT fog/edge computing, such as the block chain [4], intrusion detection system (IDS) [23], trust management scheme [24], and action-oriented programming model [9], etc. Although these security and safe mechanisms are capable of handling the most of malicious attacks, some anomalies still exist, such as the anomalies caused by hardware/software errors of fog/MEC nodes, the anomalies caused by network congestion and jitter, or the attacks which are hard to be defended such as the Denial of Service (DoS) attack [23]. To carry out the latency awareness for the CDF problem, we put forward a lightweight anomaly detection strategy. This strategy only makes use of the cumulative historical latency data of fog/MEC nodes to discovery the anomalous nodes. The results of the anomaly detection will be fed into the proposed optimization algorithm for latencies evaluation.

Many researchers modeled the energy consumption optimization problem in edge and fog computing as a mixed integer nonlinear programming problem (MINLP) [25]. This is a kind of problem for which it is difficult to find the optimal solution. Many researchers use the block coordinate descent (BCD) method to solve this kind of problem [26,27]. In these research works, the BCD method showed good performance and can quickly find the solution of complex problems. In this paper, we also use the framework of BCD method to transform the original problem into the minimum cost maximum flow sub-problem and the power control sub-problem. The proposed method is called BCDM algorithm in this paper.

In this work, we put forward a latency-aware energy-efficient **IoT-Fog-Edge Computing(IFEC)** strategy for **Continuous Data-Flow (CDF)** services. The main contributions of this work are:

- We developed a formal model for energy-efficient CDF optimization. This is a model that is composed of four level entities in IFEC computing. This model is used to formulate an optimal problem that minimizes the energy consumption subject to the latency constraints and with the anomalous fog or MEC nodes existing in systems.
- We proposed a novel lightweight anomalous nodes detection strategy for latency-aware CDF optimization.
- We designed a block coordinate descend-based max-flow algorithm to solve latency-aware energy-efficient CDF problem iteratively.
- The performance of proposed model and algorithm was evaluated by simulations based on real-life datasets.

The remainder of this paper is organized as follows: In Section 2, the motivation scenarios in E-Health system are elaborated. In Section 3 we present the system model and problem formulation. In Section 4, we put forward the proposed solutions for the CDF problem. The numerical simulation based on real-life datasets are presented in Section 5 and we draw conclusions in Section 6. The acronyms used in this paper are listed in Table 1.

## 2. Motivation Scenario

Thanks to the rapid progress of wireless communications and wearable devices, the E-Health Monitoring (EHM) System has become a paradigm of IoT applications [13,14,15,16]. A typical EHM system is composed of an IoT sensing subsystem, networking subsystem, cloud data processing and storage subsystem. In [13], the EHM system is divided into four parts, which are wearable devices, Machine-to-Machine (M2M) gateway, Network Service Capability Layer (NSCL), Data processor and openEHR services. Although the authors did not present the IoT-Fog-Edge computing, it actually can be treated as an CDF scenario because the proposed model has the same characteristics as CDF. The M2M gateways continuously collect data from sensors (such as the heart rate, blood pressure, blood oxygen saturation, etc.) and send the data periodically to data processors in the virtual machines hosted at a cloud provider. The data flow will go across the M2M gateways, NSCL and reach the cloud at last. In [14,15,16], the IoT, fog, edge and cloud integrated architecture for EHM system was clearly illustrated. The performance, including the latency, availability, and the potential challenges in EHM systems, were addressed in [13,14,15] respectively. A common characteristic in the EHM system is that the data flow starting from the IoT devices will travel across the fog layer and edge layer before it reaches the cloud data center. The network latency and availability are mainly influenced by the devices in networking subsystems such as the fog and MEC nodes. We generalized the system model of EHM system to the continuous data-flow IoT system. In our model, each IoT device has its specific domain tasks, such as the EHM tasks. The data flow generated by different IoT devices can go through same or different network paths. Our aim is to minimize the global energy consumption of multiple IoT devices while subject to the latency constraints at each IFEC level.

## 3. System Model

In CDF application scenarios, nodes in the networks could be categorized into following four levels according to their computation capability:

1. **IoT end level**: The IoT end devices belongs to this level, such as sensors, RFID, etc. which are used to collect the raw data in IoT.
2. **Fog level**: The fog nodes with very limited communication and computation capability belong to this level, such as the IoT gateway.
3. **MEC level**: The server clusters with limited computation capability belong to this level, which are deployed at places in proximity to access points of mobile networks, such as at the macro LTE base stations, at a multi-Radio Access Technology cell aggregation site, etc. [5].
4. **Cloud level**: There are only data centers at this level.

As previously mentioned, in the continuous data flow service scenarios, the IoT end devices should send the data to Fog nodes or MEC servers for preprocessing before these data are sent to data centers. A client software should be installed on end devices to communicate with the Fog nodes and MEC servers to receive the application services. These applications have domain-specific tasks that are offloaded to Fog nodes or MEC servers to execute all kinds of complicated computation, e.g., intelligent video acceleration, augmented reality (AR), etc. The computation resources are actually virtual machines that are deployed specifically for users’ applications. The VMs are referred to as *Proxy VMs* [28].

Without loss of generality, we assume the data traffic starting at the end devices should go through the fog nodes to the MEC servers and reach the data center at last. In the case that the end device does not need to offload data to the MEC server, but directly sends data to the data center by the fog node, the MEC node can be merely treated as an access point to the core networks. Our aim is to minimize the energy consumption of end devices while subject to the latency constraint in each level.

The system framework can be formulated as a tuple (UE,FN,EC,DC). UE is a collection with *I* IoT end devices. $UE=\{ue_{i},i=1,2,\ldots ,I\}$. FN is a collection with *J* fog nodes, $FN=\{fn_{j},j=1,2,\ldots ,J\}$. EC is a collection with *M* MEC nodes, $EC=\{ec_{m},m=1,2,\ldots ,M\}$. DC is the data center. Each $ue_{i}$ can be expressed as a triple tube: $ue_{i}=\left\{\lambda_{i},P_{i},\tau_{i}\right\}$, where $\lambda_{i}$ is the arrival rate of tasks of $ue_{i}$ in a time unit. We assume tasks arrive at $ue_{i}$ according to the poisson distribution. The value of arrival rates of Poisson distribution can be estimated by fitting method [29]. $P_{i}$ is the wireless transmission power of $ue_{i}$ and $\tau_{i}$ is the maximum acceptable latency for finishing a task to ensure the quality of services(QoS). $fn_{j}$ and $ec_{m}$ can be expressed as tubes $fn_{j}=\left\{\mu_{j},pr_{j}\right\}$ and $ec_{m}=\left\{\mu_{m},pr_{m}\right\}$ respectively, where μ is the service rate or processing capability, i.e. the expected number of tasks which can be processed in a time unit. We assume service time of a fog node or an MEC node follows exponential distribution with mean $\frac{1}{\mu }$. pr is the reliability that a task can be executed successfully.

### 3.1. Anomalous Nodes Discovery and Confidence Evaluation

Latency variation in IoT may be caused by network congestion or jitter. The slight variation would not change the regularity of the statistic features of network latency. On the contrary, the software or hardware errors, or attacks from the malicious users, may cause the anomalies of fog nodes and MEC nodes. Thus, the network performance of these nodes may become unstable and unpredictable. To guarantee the QoS of data flow services, an optimal solution for CDF problem should bypass these anomalous nodes. We put forward a lightweight anomalous nodes discovery strategy. This strategy is based on the observation that the anomalous nodes will exhibit some anomalous behaviors and deviate from the statistic regularity of normal nodes.

We give following definition of anomalies according to the definition of Das et al. [30].

**Definition** **1.** *In our paper, anomalies are defined as any observations of latencies that are different from the normal behavior of the latency data.*

#### 3.1.1. Chi-Square Test and Similarity Measurement

$\chi^{2}$ test is a test of the goodness-of-fit [31]. It is used to test the null hypothesis that the observed data comes from a specific distribution. Given the sample size is large enough, we have following null Hypothesis 1:

**Hypothesis** **1 (H1).** *The latency value of the observed fog or MEC node comes from the normal distribution.*

This hypothesis derives from the research on latency characteristics of mobile IoT [13]. If a fog node or MEC node is disrupted, its statistic feature of latency should be different with the normal distribution. Let $p_{i}\left(t\right)$ be the p-value got from *t*th round $\chi^{2}$ test against latency sample $\ell_{i}\left(t\right)$ of *i*th node. $\ell_{i}\left(t\right)=\left(s_{i}\left(0\right),s_{i}\left(1\right),\ldots ,s_{i}\left(t\right)\right)$ is a sample vector and $s_{i}\left(t\right)$ is the *t*th latency sample of node *i*. $\varphi_{i}\left(t\right)=\left(p_{i}\left(0\right),p_{i}\left(1\right),\ldots ,p_{i}\left(t\right)\right)$ is the vector of p-value. The Cosine similarity of latency distribution of two nodes is defined as follows:

(1) $$\begin{array}{cc} \; & sim\left(i,j\right)=\frac{\varphi_{i}\cdot \varphi_{j}}{∥\varphi_{i}∥\times ∥\varphi_{j}∥} \end{array}$$

Let the node *j* be a normal node which is selected as a *guard* by the service provider of data flow services. If the node *i* is also a normal node, it should show similar behaviors with *i* from the perspective of latencies. The similarity can be measured by the cosine function sim(i,j). Those nodes of which behaviors deviate from the baseline behaviors can be treated as anomalous nodes. The concept *guard node* is used in the anomaly detection and monitoring in wireless sensor networks (WSN) [18,32]. Guard node is a kind of node used for anomaly detection in wireless sensor networks (WSN). The guard node is between the sending node and the receiving node, which can be used for normal communication and monitoring. The selection of guard nodes is based on the location and trustworthiness of the nodes [18,33,34]. Because the continuous data flow network in this paper is not a WSN-like mobile ad-hoc network, so when selecting guard nodes, we do not need to consider the location factor, but only need to select according to the trustworthiness of the nodes. Including the architecture of nodes, security level and other trust related attributes of nodes can be used as a measurement of trustworthiness. The guard nodes in WSN should be responsible for monitoring and detecting the anomalies in addition to serving as the baseline. Nevertheless, the guard nodes in our work merely are treated as the baseline. The issues on monitoring and detecting malicious nodes are out of scopes of this work.

#### 3.1.2. F-Test

In some cases, the behaviors of a node may conform to normal distribution but the node does not have the same parameters of distributions as the guard node. An F-test is used to test whether two samples come from the normal distribution with the same variance. In this context, the variance of the latency vector of a node is compared to the one of the guard node by F-test. If the p-value of the test is less than 0.05, we reject the null Hypothesis 2.

**Hypothesis** **2 (H2).** *The latencies of the observed fog or MEC node and of the guard node come from the normal distributions with the same variance.*

#### 3.1.3. Put It All Together

According to the results from Section 3.1.1 and Section 3.1.2, we can calculate the reliability value. The real value corresponding to different reliability level depends on the application in practice. For example, 0.9 can be treated as a high value of reliability for most business applications. However, applications in finance and banking industry require more than 0.99 reliability. A simple way to estimate the real value is to calculate it by the value of the guard node *j* as follows:

(2) $$\begin{array}{cc} \; & pr_{i}=IND\left(\left\lfloor \frac{sim(i,j)}{0.5}\right\rfloor \right)\left(\frac{1}{F_{t}\left(i,j\right)}pr_{j}\right)\\ \; & \;\;+\left(1-IND\left(\left\lfloor \frac{sim(i,j)}{0.5}\right\rfloor \right)\right)sim\left(i,j\right)pr_{j} \end{array}$$

where ⌊x⌋ is to get the largest integer value less than/equal to *x*. IND(x)=1 when x≥1 and IND(x)=x otherwise. $F_{t}\left(i,j\right)$ is the F-test for node *i* and *j*. When the number of latency samples *n* approaches +∞, the degree of freedom also approaches +∞, and $F_{t}\left(i,j\right)$ should approach 1 given the node *i* is as confident as guard node *j*. It should be noted that our aim is not to get the accurate value of reliability of a node. Our aim is to find the anomalous nodes and bypass these nodes in the optimal solution of the CDF problem given there are alternative normal nodes.

**Figure 1:**
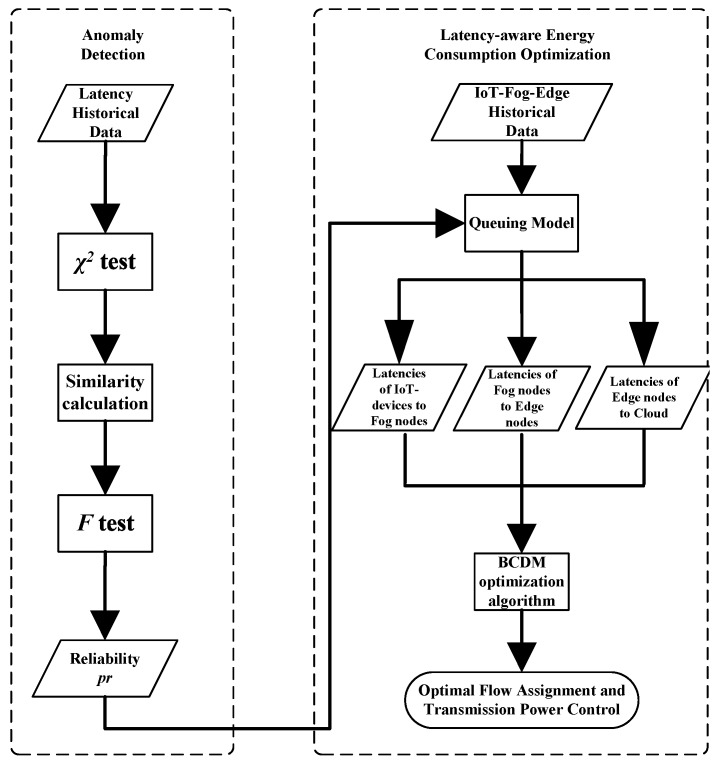
Flowchart of the optimization procedure of CDF.

Figure 1 is used to illustrate the execution process of proposed anomaly detection algorithm and its position in the whole optimization model.

### 3.2. Tandem Queue Model

**Figure 2:**
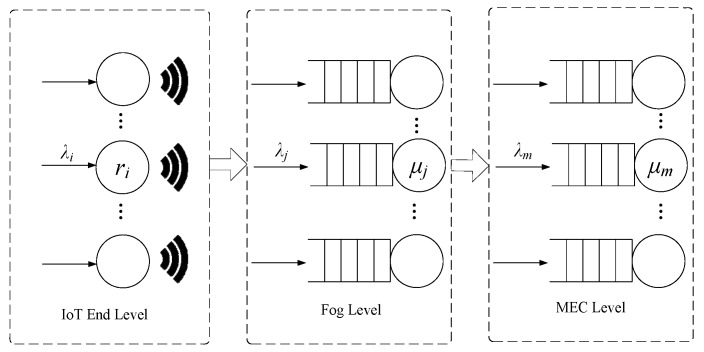
The tandem queue model of IoT-Fog-Edge Computing.

We put forward the tandem queue model depicted in Figure 2 for investigating the execution latency of tasks.

#### 3.2.1. Latency in IoT End Level

In the IoT end level, tasks arrive at end equipment $ue_{i}$ according to the poisson distribution with arrival rate $\lambda_{i}$. Without loss of generality, we assume the data size of each task is equal to *d*. The transmission latency at this level can be expressed as following equations.

(3) $$\begin{array}{cc} \; & T_{i}^{comm}=\frac{1}{\frac{r_{i}}{d}-\lambda_{i}},i=1,2,\ldots ,I \end{array}$$

(4) $$\begin{array}{cc} \; & r_{i}=B\log\left(1+\frac{P_{i}H_{i}}{\sigma^{2}}\right) \end{array}$$

where *B* is the wireless channel bandwidth. $P_{i}$ is the transmission power. $\sigma^{2}$ is the variance of complex white Gaussian channel noise. $H_{i}$ is the average channel gain. $r_{i}$ is the average transmission data rate. $\frac{r_{i}}{d}$ is the number of tasks which can be transmitted in a time unit through the wireless channel. The transmission queue of each end equipment is an M/M/1 queue. To guarantee the queue stability, following constraint must be satisfied:

(5) $$\begin{array}{c} \lambda_{i}<\frac{r_{i}}{d},i=1,2,\ldots ,I \end{array}$$

#### 3.2.2. Latency in Fog and MEC Level

Same as the model at the IoT end level, the latency in fog level and MEC level can also be expressed as the queue model as follows:

(6) $$\begin{array}{cc} \; & T^{IoT\to fog}=\frac{x_{ij}d}{r_{i}}\leq \tau_{1} \end{array}$$

(7) $$\begin{array}{cc} \; & T_{j}^{fog}=\frac{1}{pr_{j}}\left(\frac{1}{\left(\mu_{j}-\lambda_{j}\right)}\right)\leq \tau_{2} \end{array}$$

(8) $$\begin{array}{cc} \; & \mu_{j}>\lambda_{j} \end{array}$$

(9) $$\begin{array}{cc} \; & \sum_{i=1}^{I}x_{ij}=\lambda_{j},j=1,2,\ldots ,J \end{array}$$

where $x_{ij}$ is the number of tasks sent from the $ue_{i}$ to the $fn_{j}$. $\frac{x_{ij}d}{r_{i}}$ is the transmission duration that the $ue_{i}$ transmits $x_{ij}d$ units data to $fn_{j}$.

(10) $$\begin{array}{cc} \; & T_{m}^{mec}=\frac{1}{pr_{m}}\left(\frac{1}{\mu_{m}-\lambda_{m}}\right)\leq \tau_{3} \end{array}$$

(11) $$\begin{array}{cc} \; & \mu_{m}>\lambda_{m} \end{array}$$

(12) $$\begin{array}{cc} \; & \sum_{j=1}^{J}x_{jm}=\lambda_{m},m=1,2,\ldots ,M \end{array}$$

The $pr_{j}$ and $pr_{m}$ are the evaluated reliability that a task can be executed successfully by a node. As previously mentioned, we measure the confidence of the nodes in our model and map the confidence to the reliability. According to the binomial distribution, the expectation of the number of retrying for one successful execution is $\frac{1}{pr}$.

### 3.3. Problem Formulation

The latency-aware energy-efficient CDF problem can be formulated as following expression:

(13) $$\begin{array}{cc} \; & \mathrm{MP}:\underset{x_{ij},P_{i}}{Minimuze}\;U=\sum_{i=1}^{I}\sum_{j=1}^{J}E_{i}x_{ij}\\ \; & Subject\;to:\\ \; & \left(3\right)∼\left(12\right) \end{array}$$

(14) $$\begin{array}{cc} \; & x_{ij}\in \left\{0,1,2,\cdots ,x^{max}\right\} \end{array}$$

(15) $$\begin{array}{cc} \; & P_{i}\in \left[P^{min},P^{max}\right] \end{array}$$

where $E_{i}=\frac{P_{i}}{r_{i}}d$. We assume that an IoT node can only be connected to one access point in a duration. Thus, transmission power should be same for the same IoT node. So is the transmission rate. The problem **MP** is a mixed-integer-non-linear programming problem (MINLP), which is an NP-hard problem [35]. There is one multi-dimension combinatorial decision variable *x* and one continuous multi-dimension decision variable *P*. There is no generic optimal algorithm to solve this kind of problem in polynomial time complexity. In following section, we put forward two approximate algorithms, the block coordinate descent based multi-flow algorithm (BCDM) and Best-effort algorithm.

## 4. Solutions

### 4.1. Block Coordinate Descent Based Multi-Flow Algorithm

In the conventional block coordinate descent method, the vector of variables is partitioned into different blocks. The sub-function over each block with all other blocks fixed is assumed to be differentiable and convex [36,37]. Nevertheless, the original problem **MP** does not satisfy these assumptions. Thus, we have to relax the original problem **MP**. First, we fix the integer variables *x* to get the following sub-problem:

(16) $$\begin{array}{cc} \; & \mathrm{SP}1:\underset{P_{i}}{Minimuze}\;U\left(P_{i}\right)=\sum_{i=1}^{I}\sum_{j=1}^{J}\frac{P_{i}d}{r_{i}}x_{ij}\\ \; & Subject\;to:\left(3\right)∼\left(6\right) \end{array}$$

(17) $$\begin{array}{cc} \; & P_{i}\in \left[P^{min},P^{max}\right] \end{array}$$

where constraints (4)–(6) can be further reduced to following expression:   

(18) $$\begin{array}{cc} \; & P_{i}\geq \frac{\left(2^{\frac{x_{ij}d}{B\tau_{1}}}-1\right)\sigma^{2}}{H_{i}},j=1,\cdots ,J \end{array}$$

The objective function of **SP1** is not meant to be convex. However, according to the research in [37], if a function *F* is differentiable and strictly quasi-convex over each block, its limit point is a critical point. Thus, we only need to confirm that $U\left(P_{i}\right)=\sum_{i=1}^{I}\sum_{j=1}^{J}\frac{P_{i}d}{r_{i}}x_{ij}$ is quasi-convex. We have following proposition:

**Proposition** **1.** *Let $F\left(P_{i}\right)=\frac{P_{i}}{r_{i}}$, F is quasi-convex. Where $P_{i}$ and $r_{i}$ are the transmission power and data rate respectively.*

**Proof.**  At first, we calculate the first order derivative of *F*.

$$\begin{array}{cc} \; & \frac{dF}{dP_{i}}=\frac{\ln2}{\ln\left(aP_{i}+1\right)}-\frac{aP_{i}\ln2}{\left(aP_{i}+1\right)\ln^{2}\left(aP_{i}+1\right)} \end{array}$$

(19) $$\begin{array}{cc} \; & \;\;=\frac{\ln2}{\ln\left(aP_{i}+1\right)}\left(1-g\left(P_{i}\right)\right) \end{array}$$

(20) $$\begin{array}{cc} \; & g\left(P_{i}\right)=\frac{aP_{i}}{\left(aP_{i}+1\right)\ln\left(aP_{i}+1\right)} \end{array}$$

where $a=\frac{H_{i}}{\sigma^{2}}$. $aP_{i}$ is actually the signal-to-noise ratio (SNR). Let $aP_{i}+1=x$, $g\left(P_{i}\right)\Rightarrow g^{\prime }\left(x\right)=\frac{x-1}{x\ln x}$. We have the following result:

(21) $$\begin{array}{cc} \; & \forall a>0,\lim_{P_{i}\to 0}\;g\left(P_{i}\right)=\lim_{x\to 1}\;g^{\prime }\left(x\right)=\lim_{x\to 1}\;\frac{x-1}{x\ln x} \end{array}$$

(22) $$\begin{array}{cc} \; & \;\;\;\;\;\;\;\;=\lim_{x\to 1}\;\frac{1}{\ln x+1}=1 \end{array}$$

Then we calculate the first order derivative of $g(P_{i})$:

(23) $$\begin{array}{c} \frac{dg(P_{i})}{dP_{i}}=\frac{a\left(\ln\left(aP_{i}+1\right)-aP_{i}\right)}{{\left(aP_{i}+1\right)}^{2}\ln^{2}\left(aP_{i}+1\right)} \end{array}$$

$\frac{dg(P_{i})}{dP_{i}}<0$ when $P_{i}>0$, thus $g(P_{i})$ is **monotonic decreasing**. Hence according to (21) and (22), $g(P_{i})<1$ will always hold with the $P_{i}>0$. Thus, $\frac{dF}{dP_{i}}>0$ always holds too, which means **$F(P_{i})$ is monotonic increasing**. Suppose $P_{1}$ and $P_{2}$ are two transmission power values, $P_{1}\geq P_{2}$. ∀α∈[0,1], we can get following result:

(24) $$\begin{array}{cc} \; & F\left(\alpha P_{1}+\left(1-\alpha \right)P_{2}\right)\leq F\left(\alpha P_{1}+\left(1-\alpha \right)P_{1}\right)\\ \; & \;\;\;\;\;\;\;\;=F\left(P_{1}\right)=max\left(F\left(P_{1}\right),F\left(P_{2}\right)\right) \end{array}$$

This result means $F(P_{i})$ is quasi-convex. □

According to the Proposition 1, the $U(P_{i})$ is also quasi-convex because it is a polynomial of $F(P_{i})$.

Then we consider the sub-problem in which continuous variables are fixed. The sub-problem can be expressed as follows:

(25) $$\begin{array}{cc} \; & \mathrm{SP}2:\underset{x_{ij}}{Minimuze}\;U=\sum_{i=1}^{I}\sum_{j=1}^{J}\frac{P_{i}d}{r_{i}}x_{ij}\\ \; & Subject\;to:\left(7\right)∼\left(12\right) \end{array}$$

(26) $$\begin{array}{cc} \; & x_{ij}\in \left\{0,1,2,\cdots ,x^{max}\right\} \end{array}$$

The **SP2** is an integer programming problem, which is not convex or quasi-convex. Hence we have to transform it to a convex problem.

Constraints (7)∼(12) can be transformed as follows:

(27) $$\begin{array}{cc} \; & 0\leq x_{ij}\leq \tau_{1}\frac{r_{ij}}{d} \end{array}$$

(28) $$\begin{array}{cc} \; & 0\leq x_{jm}\leq \mu_{j}-\frac{1}{\tau_{2}pr_{j}} \end{array}$$

(29) $$\begin{array}{cc} \; & 0\leq x_{mt}\leq \mu_{m}-\frac{1}{\tau_{3}pr_{m}} \end{array}$$

(30) $$\begin{array}{cc} \; & \underset{j=1}{\sum^{J}}\;x_{ij}=\lambda_{i} \end{array}$$

(31) $$\begin{array}{cc} \; & \underset{i=1}{\sum^{I}}\;x_{ij}=\underset{m=1}{\sum^{M}}\;x_{jm} \end{array}$$

(32) $$\begin{array}{cc} \; & \underset{j=1}{\sum^{J}}\;x_{jm}=x_{mt}\\ \; & i=1,\cdots ,I;j=1,\cdots ,J;m=1,\cdots ,M;t=1 \end{array}$$

where the index variable *t* represents the data center. This is a kind of linear relaxation. After the linear relaxation, the **SP2** can be solved by linear programming solver in polynomial time-complexity steps. Nevertheless, the solution cannot be ensured to be integral. To guarantee the solution is integral, following transformation should be carried out.

**Figure 3:**
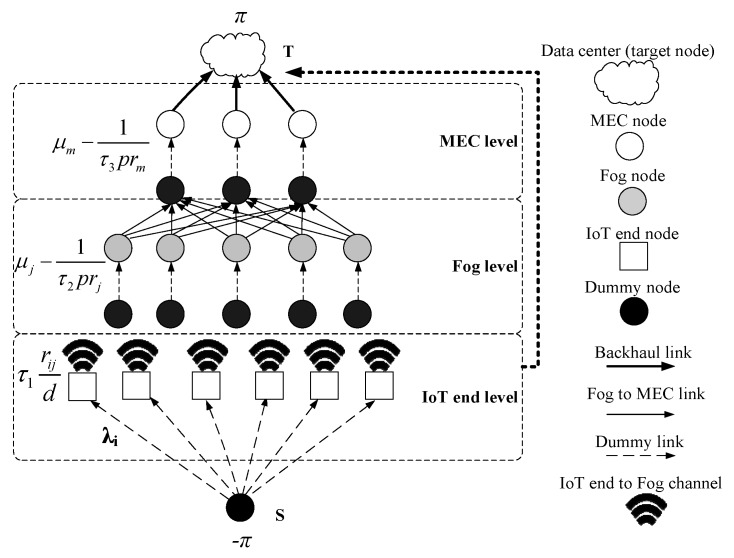
The Minimum Cost Maximum Flow Model for the CDF problem in IFEC.

As depicted in Figure 3, a dummy node **S** is added to the IFEC model as a source node, which has some dummy edges to be connected with the IoT end nodes. Each fog node or MEC node are split into two nodes connected by a dummy edge with a weight, which is the upper bound of processing capability of the corresponding node. A dummy edge connecting the **S** with **T** is added. If some tasks could not be offloaded to fog nodes, they will be sent to **T** directly by this dummy edge. This dummy edge has almost infinite throughput(such as the 5G communication channel) but very high energy consumption. If a task went through this dummy edge, it means the failure of receiving fog computing service and it is not a part of solution. Thus, the CDF model can be converted to a Minimum Cost Maximum Flow model (MCMF). To guarantee that the solution is integral, the parameters should be integers, which can be achieved by rounding and scaling. For example, if the capacity of a node is 4.2, it can be rounded to 4. If the energy consumption is 0.3 Joule, it can be scaled up to 300 Millijoule.

After the above linear relaxation and transformation are conducted, the original problem **MP** was converted to two convex (or quasi-convex) sub-problems, thus it can be solved by block coordinate descent algorithm iteratively. The pseudo codes of Block Coordinate Descent-based Multi-flow algorithm (BCDM) is illustrated in Algorithm 1.

Based on the above analysis, we can get the following conclusions about the effectiveness of BCDM algorithm:

Since every subproblem of CDF optimization is differentiable, convex and strictly quasi-convex, the BCDM algorithm can converge to the local optimal solution.

After the linear relaxation of the subproblem SP2, we use the simplex method to solve the linear programming problem. Because the worst-case time complexity of the simplex method is exponential, so the worst-case time complexity of the BCDM algorithm is exponential. However, in general, simplex algorithm is an efficient algorithm, which can get the solution in polynomial time. Therefore, in general, BCDM algorithm is polynomial time complexity algorithm.
**Algorithm 1** Block Coordinate Descent based Multi-flow Algorithm**Input:** $\lambda_{i},\mu_{j},\mu_{m},\tau_{1},\tau_{2},\tau_{3},H_{i},B,\sigma ,pr_{j},pr_{m}$;**Output:** x,P;1:Initialize $P_{i}$ as $P^{min}$2:**while**$U^{old}-U^{new}>\varepsilon$**do**3:    solve MCMF problem with $P_{i}$ fixed to get $x_{ij}$4:    update each $P_{i}$ iteratively with $x_{ij}$ and the other $P_{j}$ fixed, i≠j5:    renew *U*, *P* if $U^{new}$ descends6:**end while**7:**return**x,P

### 4.2. Best-Effort Algorithm

Since there is no same work currently to solve the CDF problem as proposed in this paper, we use the Best-effort method as the benchmark, which was used for IP data-flow service in multi-access wireless networks and can be adapted to the CDF problem easily. The Best-effort algorithm is carried out on the different level. At each level, the algorithm always tries its best to allocate the tasks to one node in the next level till it reached its upper capacity, and then turn to another node in the next level. The Best-effort algorithm is illustrated in Algorithm 2.
**Algorithm 2** Best-effort Algorithm**Input:** $\lambda_{i},\mu_{j},\mu_{m},\tau_{1},\tau_{2},\tau_{3},H_{i},B,\sigma ,pr_{j},pr_{m}$;**Output:** x,P;1:**for** each *i* in *I* **do**2:    **for** each *j* in *J* **do**3:        allocate as many tasks of $ue_{i}$ as possible to $fn_{j}$ till reach its capacity4:    **end for**5:    calculate $P_{i}$ for each $ue_{i}$6:**end for**7:**while** the solution is infeasible **do**8:    carry out the Best-effort strategy in fog level9:    carry out the Best-effort strategy in MEC level10:    if the solution is infeasible, decrease the $x_{ij}$ with largest $P_{i}$ unless $x_{ij}$ becomes 011:**end while**12:**return**x,P

## 5. Simulations

In this section, two types of simulations are presented. One was conducted for evaluating the feasibility of proposed latency awareness strategy, the other was conducted for measuring the performance of proposed BCDM algorithm.

### 5.1. Anomaly Detection Based Latency Awareness

According to the study by Pereira et al. [13], the E-Health Monitoring (EHM) ecosystem can be divided into Gate Way (GW), Network Service Capability Layers (NSCL), Data Processor (DP) and openEHR services. They reported their real-life experiment results on evaluating the latency performance of service composition of a mobile E-Health application. *They argued that end-to-end (E2E) latency is composed of latencies between neighbor system components and the latencies that compose the E2E latency follow a normal distribution*. Part of the measured latency values are listed in Table 2.

To test the proposed strategy of anomaly detection based reliability evaluation, we generated a sample sequence of latencies according to the normal distribution and the latency between GW and NSCL in Table 2. This sample sequence was chosen as the baseline of the anomaly detection. The other two sample sequences also were generated as the historical data of anomalous nodes. One of the sequences was the latencies of the malicious node, and generated using the same way as generating the baseline except that some anomalous data were injected into the sequence. We assumed that the malicious node would misbehave with 50% probability [38], thus at each sampling point, the malicious node may generate the normal latency values with 50% probability. In other 50% probability cases, the sample generation is failure. The sample generation will be retried with adding a waiting duration to the latency. The maximum number of retries is 4, and the waiting duration is 1, 2, 4, 8 respectively. The final latency will be set as 1000 s if the maximum retries reached. Another sample sequence is the latencies of the abnormal node. The abnormal node here refers to the node that has not been maliciously attacked although it shows abnormal behavior. This sequence was generated according to the normal distribution but with different distribution parameters. Total 1000 samples were generated for each sample sequence. The order of the samples in the sequence was treated as the rounds of consecutive sampling in the real scenario.

**Figure 4:**
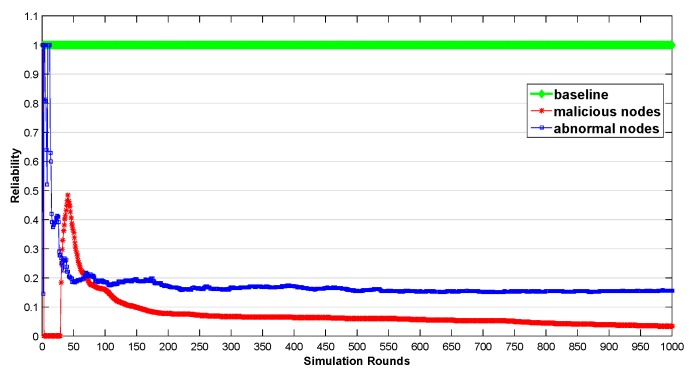
The Reliability Evaluation in 1000 Rounds Consecutive Sampling.

The simulation results are shown in Figure 4. The reliability was calculated according to Equation (1). The reliability of the baseline node is always 1. As shown in the results, the reliability of the malicious node and abnormal node fluctuated obviously in the first 50 rounds. This is because the proposed scheme is based on the central limit theorem of probability statistics. The central limit theorem works only when the number of samples is large enough. After 50 rounds, the reliability of malicious and abnormal nodes decreased gradually. Finally, the reliability of abnormal nodes was gradually stable in the interval between 0.1 and 0.2, while that of malicious nodes approach 0. Experimental results show that our scheme can effectively detect anomalous nodes. It should be noted that our purpose is not to get the real probability of normal behavior of anomalous nodes, but to get a reliable estimate value, and use this estimate value to select normal nodes first, and bypass anomalous nodes.

### 5.2. Energy Efficient Optimization for IoT Continuous Data-Flow Services

The effective of the proposed energy efficient optimization algorithm was verified in this section.

#### 5.2.1. Task Arrival Rate Analysis

He et al. [29] studied the traffic arrival mode of the GSM base station and verified that it conforms to the Poisson distribution. The task arrival rate used in this section was set according to the real dataset provided by Barlacchi et al. [39]. There are several different datasets in their research, such as telecommunications, weather, news, social networks, etc. In our simulation, we focused on the Internet traffic activity data. Their dataset contains data collected from Milan city and province of Trentino. The areas of Milan and Trentino are divided into grids, which are composed of squares with size of about 235×235 m. Each square is assigned a square ID. The data set contains the amount of traffic reached in unit time (10 min) in each square. We used Poisson parameter estimates to fit the arrival rate per hour, and got the confidence interval of the hourly arrival rate with 95% confidence in one day.

**Figure 5:**
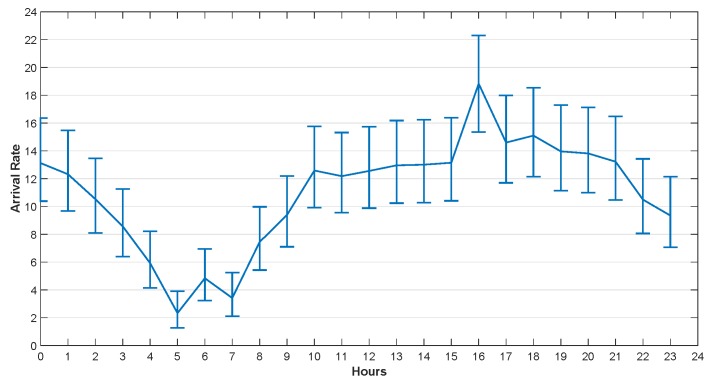
The Arrival Rate in 24 h, Square id 1000, Trentino, 1 November 2013.

**Figure 6:**
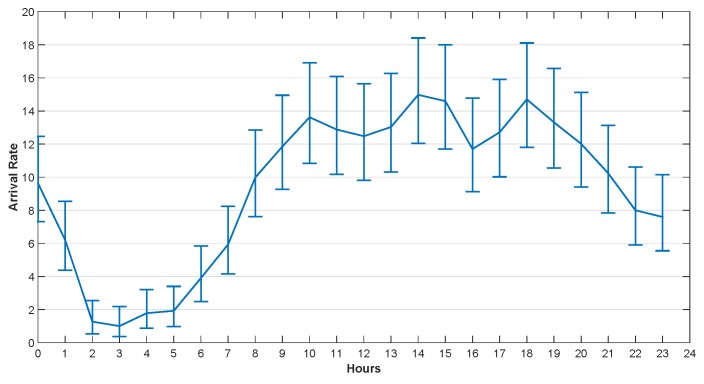
The Arrival Rate in 24 h, Square id 1, Milan, 1 November 2013.

**Figure 7:**
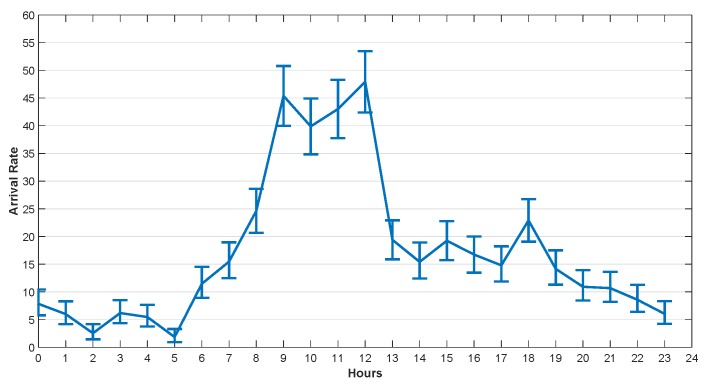
The Arrival Rate in 24 h, Square id 100, Milan, 1 November 2013.

**Figure 8:**
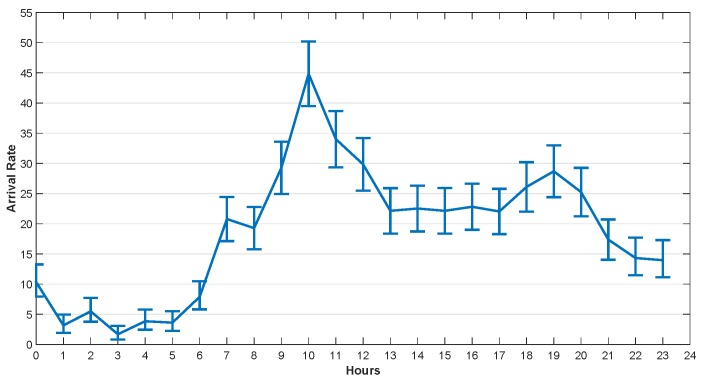
The Arrival Rate in 24 h, Square id 10000, Milan, 1 November 2013.

The arrival rates in 24 h at 4 different squares in Trentino and Milan are illustrated in Figure 5, Figure 6, Figure 7 and Figure 8. The curves drawn in these figures are the average of the arrival rate, and the error bars represent the upper and lower bounds of the confidence interval. As shown in the figures, the arrival rate will vary greatly in different hours and in different regions. The minimum value of arrival rate is less than 2, while the maximum value is close to 55. It should be noted that in [39], every time a user started an Internet connection, a record will be generated. However, it is well known that the amount of data traffic generated by each connection is different. In addition, during the connection, the user program may generate multiple tasks, and the size of each task is different too. For example, the data size of sending an instruction or a heartbeat signal does not exceed 1 KB, while the data size of sending a face recognition picture may exceed 5 MB. Therefore, for the convenience of analysis, we consider that each CDF task has the same size and does not exceed the maximum transmission unit (MTU) of TCP/IP protocol, i.e., 1500 bytes. For the records in [39], suppose 1 MB data was generated for each user connection. If the arrival rate was 10 within one hour, the data size generated was 10 MB. That was equivalent to 7000 tasks arriving in an hour in our paper.

#### 5.2.2. Verification of the Proposed Algorithm

Some of the simulation parameters in this section are listed in Table 3. The performance metrics are total energy consumption and average energy consumption (AE) of IoT end nodes. AE is calculated as following equation:

(33) $$\begin{array}{cc} \; & AE=\frac{U}{\sum_{i=1}^{I}\sum_{j=1}^{J}x_{ij}} \end{array}$$

**Figure 9:**
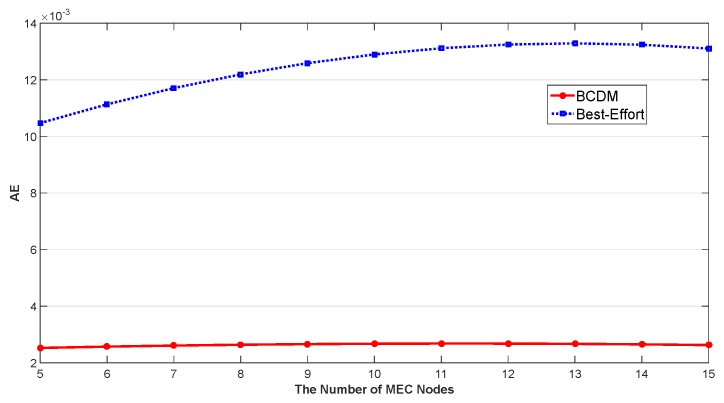
The Impact of the Number of MEC Nodes to Average Energy Consumption.

**Figure 10:**
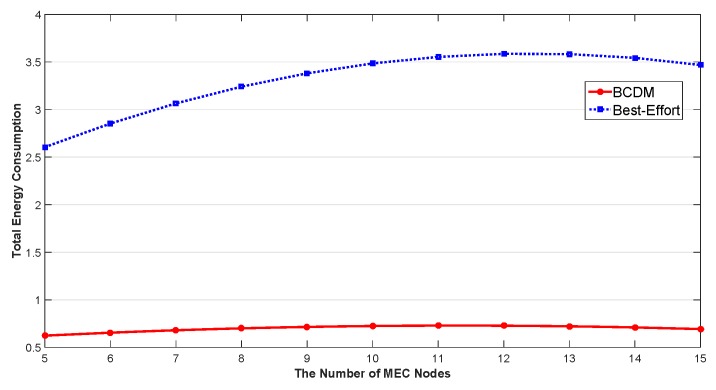
The Impact of the Number of MEC Nodes to Total Energy Consumption.

**Figure 11:**
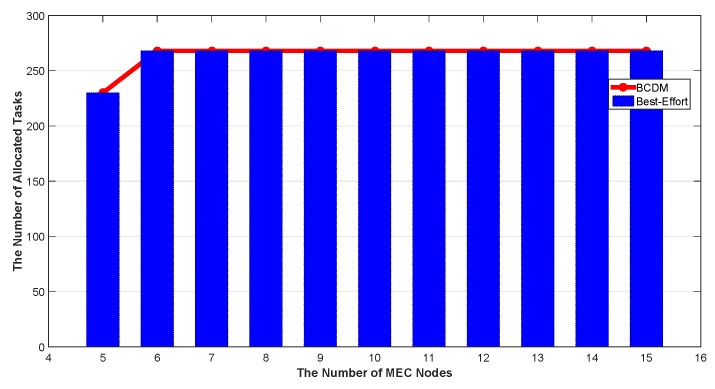
The Impact of the Number of MEC Nodes to Total Number of Allocated Tasks.

**Figure 12:**
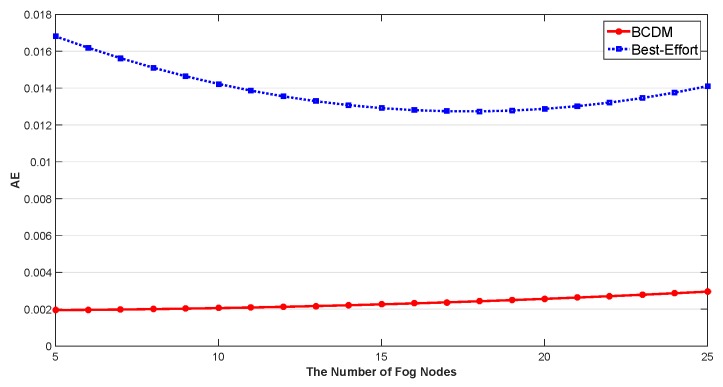
The Impact of the Number of Fog Nodes to Average Energy Consumption.

**Figure 13:**
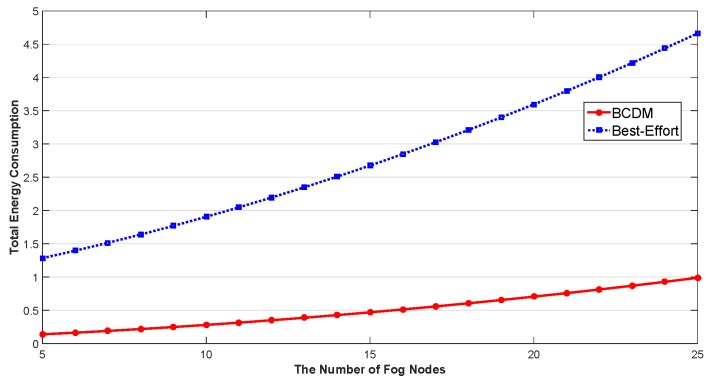
The Impact of the Number of Fog Nodes to Total Energy Consumption.

**Figure 14:**
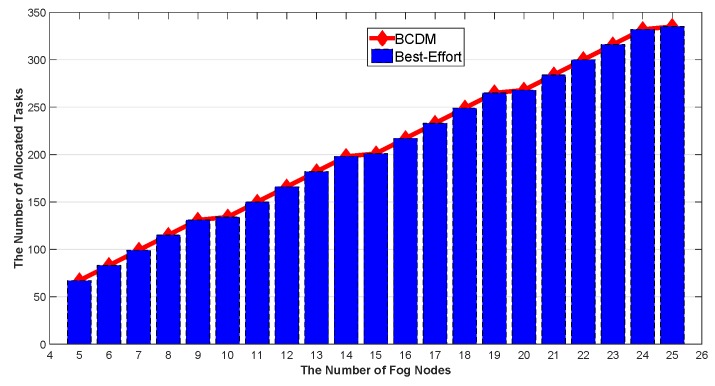
The Impact of the Number of Fog Nodes to Total Number of Allocated Tasks.

**Figure 15:**
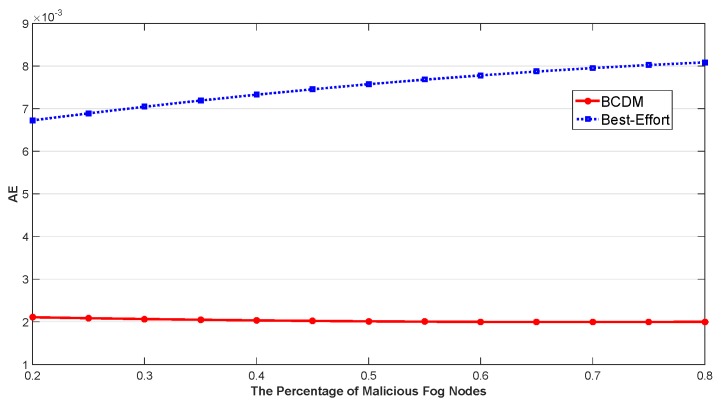
The Impact of Percentage of Malicious Fog Nodes to Average Energy Consumption.

**Figure 16:**
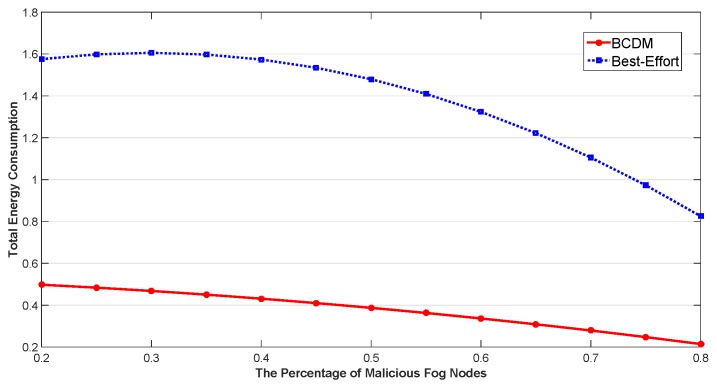
The Impact of Percentage of Malicious Fog Nodes to Total Energy Consumption.

**Figure 17:**
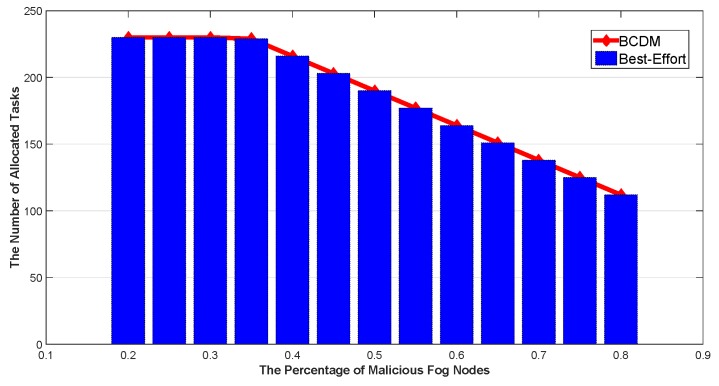
The Impact of Percentage of Malicious Fog Nodes to Total Number of Allocated Tasks.

We studied the impact of the number of MEC nodes, the impact of the number of fog nodes and the impact of percentage of malicious fog nodes in this section. The results are shown in Figure 9, Figure 10, Figure 11, Figure 12, Figure 13, Figure 14, Figure 15, Figure 16 and Figure 17 respectively. As shown in the results, both BCDM algorithm and Best-effort algorithm showed good scalability when the simulation parameters changed, especially in the presence of malicious nodes. This is because the latency-awareness strategy based on anomaly detection proposed in this paper can effectively bypass the anomalous nodes, thus reducing energy consumption. In all cases, BCDM algorithm showed better performance than Best-effort algorithm. It should be noted that a line chart and a bar chart are used to show the number of tasks allocated in Figure 11 and Figure 14. This is because the number of tasks assigned by BCDM and Best-effort algorithm is the same, and different graphical methods are used to show them more clearly.

The impact of the number of fog nodes and MEC nodes on energy consumption is shown in Figure 9, Figure 10, Figure 11, Figure 12, Figure 13 and Figure 14. As depicted in these figures, the impact of the number of fog nodes on energy consumption was greater than that of MEC nodes. This is because in our simulation parameter setting, MEC node has stronger task processing ability, which is also one of the main differences between fog nodes and MEC nodes. Therefore, in the simulation, the main bottleneck of performance was exhibited in the fog node layer. From the simulation results, it can be found that the change of MEC node number had little effect on energy consumption, while the increase of fog node number made the decrease of energy consumption very obvious. Figure 15, Figure 16 and Figure 17 show the impact of the percentage of malicious fog nodes in all fog nodes on energy consumption. Obviously, the increase of the proportion of malicious fog nodes and the increase of the number of fog nodes have the opposite effect on energy consumption.

## 6. Conclusions

In this work, we put forward a latency-aware energy-efficient continuous data-flow optimization strategy. This strategy is designed for continuous data flow applications in IoT-fog-edge computing scenarios. The most typical application of continuous data-flow is E-health Monitoring System. We made use of a novel lightweight anomaly detection strategy to get the confidence of the fog and MEC nodes. We used the confidence as the metric to evaluate the reliability of each nodes and use it to estimate the latencies in the energy-efficient continuous data-flow problem with latency constraints. We established a formal model and solved the problem using the block coordinate descend max-flow (BCDM) algorithm. The real-life datasets were used in the numerical study to verify the performance of the proposed strategies. Numerical results showed that the proposed strategies have good performance in all simulations.

In this paper, we only consider the latency property of data flow service. However, some other network attributes will also have a great impact on the overall performance of the system, such as frequency of messages, message rates, size, etc. We will combine these attributes with latencies for measuring the system performance in our future work. Although the main motivation scenario of the continuous data flow problem in this paper is E-health monitoring system, the continuous data flow problem can also be extended to more application scenarios, such as mobile social network [40], intelligent industrial monitoring system [41], etc. We will further consider the location of fog nodes and MEC nodes in continuous data flow problem [42]. Furthermore, we will conduct more simulations in an event-driven simulator [43], such as the YAFS [44], in our future work.

## Figures and Tables

**Table 1:** Notations.

Symbol	Description
*U*	Utility function
*I*	the amount of IoT-End nodes
*M*	the amount of MEC nodes
*J*	the amount of Fog nodes
λ	arrival rate of tasks
τ	latency constraint
μ	service rate of fog and MEC nodes
*B*	bandwidth of wireless channel
*H*	channel gains
$\sigma^{2}$	Gaussian white noise

**Table 2:** Latencies In EHM System.

Parameter Name	Parameter Value (Unit)
Latency between GW and NSCL	0.9671sec±0.0186
Latency between NSCL and DP	$0.0138sec\pm 1.802\times 10^{-4}$
Latency between DP and EHR	0.3130sec±0.0470

**Table 3:** Simulation Parameters.

Parameter Name	Parameter Value (Unit)
The Distance from IoT-End Nodes to Access Points	100–500 m
The Default Number of IoT-End Nodes	60
The Default Number of Fog Nodes	20
The Default Number of MEC Nodes	10
Bandwidth of Wireless Channel	$1.08\times 10^{6}$ Hz
Default Processing Capacity of MEC Nodes $\mu_{m}$	50/sec
Default Processing Capacity of Fog Nodes $\mu_{j}$	20/sec
The Data Size of a Task *d*	$3.0\times 10^{5}$ Bit
Latency Constraint τ	0.3 s
Default Task Arrival Rate of Each IoT-Fog Node λ	10/sec
The Reliability of Normal Fog Nodes	[0.9,0.99]
The Reliability of Normal MEC Nodes	[0.9,0.99]
Default Percentage of Malicious Fog Nodes	20%
Default Percentage of Malicious MEC Nodes	10%
Default Reliability of Malicious Fog Nodes	0.2
Default Reliability of Malicious MEC Nodes	0.2
Background Noise $\sigma^{2}$	−100 (dBm)
